# Locoregional control and toxicity after pleurectomy/decortication and intensity‐modulated pleural radiation therapy in patients with malignant pleural mesothelioma

**DOI:** 10.1111/1759-7714.13668

**Published:** 2020-10-08

**Authors:** Oscar Arrieta, Francisco Lozano‐Ruiz, Monika Blake‐Cerda, Rodrigo Catalán, Luis Lara‐Mejía, Miguel Ángel Salinas, Federico Maldonado‐Magos, José F. Corona‐Cruz

**Affiliations:** ^1^ Thoracic Oncology Unit Instituto Nacional de Cancerología (INCan) México City Mexico; ^2^ Radiation Oncology Department Instituto Nacional de Cancerología (INCan) México City Mexico; ^3^ Thoracic Surgery Department Instituto Nacional de Cancerología (INCan) México City Mexico

**Keywords:** Intensity‐modulated radiotherapy, mesothelioma, multidisciplinary treatment, pleurectomy/decortication, radiotherapy

## Abstract

**Background:**

Treatment of malignant pleural mesothelioma (MPM) represents a major challenge for oncologists. Multimodality treatment, which generally involves induction chemotherapy, surgery and radiotherapy have recently shown promising results. The aim of this study was to evaluate the locoregional control and toxicity of intensity modulated radiotherapy (IMRT) after pleurectomy and decortication (P/D) as part of trimodality therapy for patients with locally advanced MPM.

**Methods:**

We prospectively analyzed data from 20 patients with MPM treated at a single tertiary‐care institution. Initially every patient received induction chemotherapy with platinum‐based chemotherapy. After chemotherapy, patients without progression underwent P/D, and if feasible, hemi‐thoracic IMRT was administered at a planned dose of 50.4–54 Gy in 28–30 fractions and treated with 9–11 noncoplanar fields.

**Results:**

A total of 15 of the 20 enrolled patients underwent P/D followed by IMRT to the hemi‐thoracic cavity. The median total radiotherapy dose was 48.7 Gy (23.4–54 Gy). Radiation pneumonitis (RP) developed in nine patients (60%), and of these, two patients (13.3%) experienced G3 or G4 RP. The estimated locoregional‐relapse‐free survival at two years was 75.9%, and the main pattern of recurrence was distant (72.7%). For the entire cohort median follow‐up was 22.7 months, median progression‐free survival was 18.9 months and median overall survival 23.6 months.

**Conclusions:**

Platinum‐based chemotherapy followed by lung‐sparing surgery (P/D) and IMRT is a feasible and safe treatment modality that yields acceptable locoregional control in patients with locally advanced MPM; however, these results should be corroborated in larger studies.

## Graphical abstract

For patients with malignant pleural mesothelioma a less invasive surgical approach than EPP, such as P/D followed by high doses of radiation, in the form of IMRT, could lead to a high rate of locoregional control and acceptable toxicity.

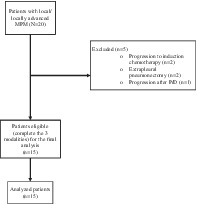

## Introduction

Malignant pleural mesothelioma (MPM) is a rare, but aggressive neoplasm with a deleterious prognosis. During the last decades, multimodality treatment has improved outcomes for patients with MPM.^1^ However, the prognosis remains poor for patients that develop this tumor. Trimodality therapy, which includes induction chemotherapy, followed by surgery and radiotherapy, has shown promising results in patients with locally and locally advanced MPM.^2^, ^3^ It must be emphasized that the outcome of the MARS trial failed to demonstrate any benefit from extrapleural pneumonectomy (EPP) within trimodal therapy over chemotherapy alone, and these results were consistent for OS, as well as for quality of life. Furthermore, more treatment‐related deaths occurred in patients that underwent EPP.^4^ Since the results of the MARS study were published, less invasive surgical techniques, such as lung‐sparing surgery with P/D have gained a lot of interest and are being increasingly used.

On the other hand, radiation to the whole hemithorax after lung‐sparing surgery is challenging considering the increased risk of toxicity occurring with two intact radiosensitive lungs.^5^ Prior evidence supports the use of hemithoracic IMRT after P/D in terms of safety and efficacy.^6^

Furthermore, IMRT after P/D compared to IMRT followed by EPP has demonstrated superiority in OS and PFS, although at the cost of decreased pulmonary function after therapy.^7^ The IMPRINT study also tested the safety and effectiveness of trimodality therapy (induction chemotherapy, P/D and IMRT) and reported an acceptable toxicity profile with few patients developing grade 3–4 pneumonitis.^1^

It must be emphasized that information regarding IMRT as a component of trimodality treatment for MPM is scarce; accordingly, the aim of the present study was to prospectively determine the locoregional control rates, and associated toxicity of trimodality therapy in patients who had undergone neoadjuvant chemotherapy followed by P/D and hemithoracic IMRT.

## Methods

### Patients

We designed a prospective study in which we enrolled patients with a confirmed diagnosis of MPM who received treatment at our institution between October 2011 and May 2016. Each patient provided their written informed consent to participate in the study. The entire protocol was approved by local scientific and bioethical committees, and was performed in accordance with the Declaration of Helsinki and the principles of good clinical practice.

Inclusion criteria were: patients ≥18 years; Karnofsky performance status (KPS) of ≥70% or ECOG PS ≤2; pathological confirmation of MPM (any histology); absence of metastatic disease; chemotherapy naïve patients; no prior radiotherapy treatment; and appropriate hematological, renal, and hepatic function tests.

### Treatment

All patients received induction platinum‐based chemotherapy. Three distinct chemotherapy regimens were considered at the discretion of the attending medical oncologist:

1. Gemcitabine 250 mg/m^2^ in a six‐hours infusion plus cisplatin 35 mg/m^2^ on days 1 and 8 of a 21‐day cycle.
2. Pemetrexed 500 mg/m^2^ in combination with cisplatin 75 mg/m^2^ or carboplatin AUC = 5 on day 1 of a 21‐day cycle.
3. Vinorelbine 25 mg/m^2^ IV on days 1, 8, 15, and 22 of a 28‐day cycle with cisplatin 100 mg/m^2^ on day 1.

Standard supportive medications in patients treated with pemetrexed‐based regimen were provided, including folic acid supplementation 400 mcg daily and vitamin B12. Dose adjustments were permitted depending on the severity of toxicities according to current guideline recommendations. Patients without documented progression after induction chemotherapy were eligible for surgical resection. The grading system used for adverse events (including pneumonitis) was based on criteria listed in the Common Terminology Criteria for Adverse Events (CTCAE; version 4.03).

### Surgery

After completion of induction chemotherapy all patients were evaluated by a thoracic surgeon to determine if lung‐sparing surgery (P/D) was feasible four to six weeks after the last cycle of chemotherapy. The surgery procedure performed (selected among three variations of P/D), was decided at the discretion of the surgeon. Extended P/D included the removal of all gross tumor along with resection of the diaphragm and/or pericardium, P/D included the removal of all gross tumor along with parietal and visceral pleurectomy but without diaphragm or pericardial resection, partial pleurectomy included partial removal of parietal and/or visceral pleura along with resection of residual gross tumor. Lung‐sparing techniques were performed in line with the recommendations of the International Association for the Study of Lung Cancer International Staging Committee.

### Radiotherapy

After surgery, all eligible patients were evaluated by the radiation oncology team before starting radiotherapy to rule out signs of progression, and to corroborate all of the following criteria: KPS >70%, no O_2_ dependence, and no signs of surgery‐related complications. In patients that fulfilled predetermined criteria, hemithoracic pleural IMRT was started 6–8 weeks after surgery, the dose prescribed for radiotherapy was between 50.4–54 Gy in 28–30 fractions and treated with 9–11 noncoplanar fields.

**Figure 1:**
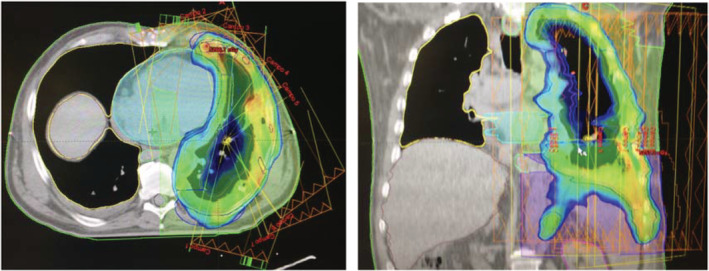
Hemithoracic IMRT treatment, in nine noncoplanar fields were used to achieve high dose in the hemithoracic cavity and lowering dose to the intact remaining lung.

A three‐dimensional CT scan was performed for simulation purposes and all patients were immobilized with a vacuum cushion, and planned using a Varian Eclipse V.11.0. Treatment was administered on a Varian Clinac iX (Fig 1). No respiratory motion techniques were implemented. The clinical target volume (CTV) was defined as the entire pleural surface, surgical clips, and any other potential site with residual microscopic disease. CTV is generally limited by thoracic inlet superiorly, the insertion of the diaphragm inferiorly, the ribs laterally, and the mediastinal pleura, pericardium, and hilum medially. Ipsilateral hilum lymph nodes were included and mediastinal lymph nodes were spared. The planning target volume (PTV) was generated using a uniform 5 mm margin around the CTV. IMRT was delivered with 6 MV, 9–11 noncoplanar fields around the ipsilateral lung. Dose constraints in the contralateral lung included a mean lung dose <3.5 Gy or V7 < 20%, and mean esophageal dose <30 Gy; the heart V15 was <40% for right sided‐tumors, and V18 < 70% in left‐sided; maximum dose to the spinal cord was <22 Gy. Liver constraints were V17 < 60%, for right‐sided tumors and V8 < 30% in left‐sided. Finally, mean dose to the kidney was <5 Gy or V7 < 33% (Table S1). The aforementioned dose constraints were respected in every patient. No dose escalation using a simultaneous integrated boost (SIB) was delivered to any patient. Patients were evaluated by CT scan 4–6 weeks after radiotherapy and subsequently every three months.

### Statistical analysis

For descriptive purposes, continuous variables were summarized as arithmetic means and standard deviations (SD); categorical variables were comprised as frequencies and proportions. The OS and PFS were analyzed by the Kaplan‐Meier method, and comparisons among subgroups were analyzed by the log‐rank test. All variables were dichotomized for the survival analyses. Statistical significance was determined as *P* ≤ 0.05 using a two‐tailed test. SPSS software version 26 (SPSS Inc., Chicago, IL) was used for all statistical analyses.

## Results

**Figure 2:**
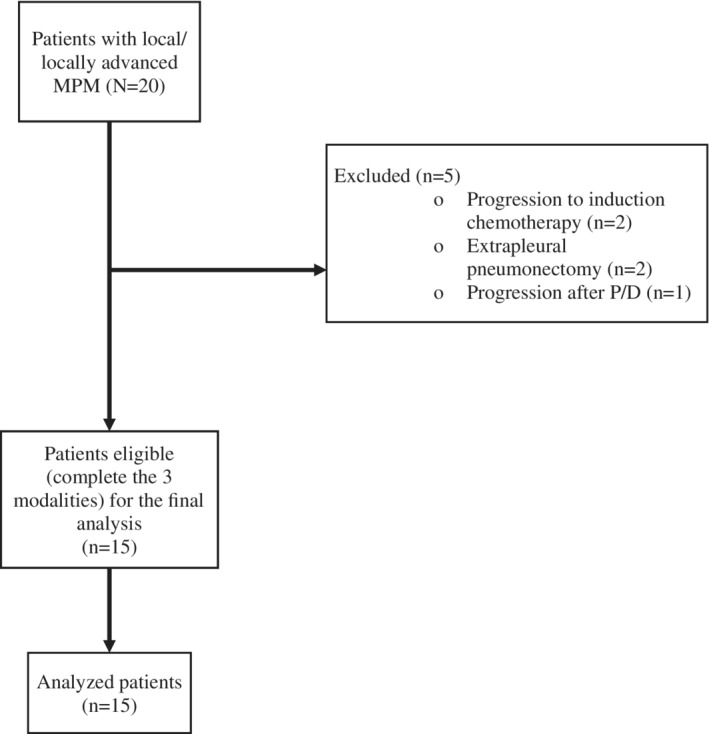
Flow‐diagram of enrolled patients.

A total of 20 patients were initially eligible to receive trimodality therapy; of these, five patients were determined not suitable to receive trimodality therapy due to disease progression during induction chemotherapy, or because they were not candidates for P/D and/or IMRT (Fig 2). The baseline characteristics of the 15 patients that received all three treatment modalities are listed in Table 1. The median age at diagnosis was 58 years, most of the patients were male (86.7%), ECOG PS 0–1 was documented in 93.3% of patients and the median KPS was 90%. The most common histology was epithelioid which was diagnosed in 93.3% of patients. The median follow‐up time was 22 months (95% CI 7.5–44.7), which was estimated by the Kaplan‐Meier method.

**Table 1:** Baseline characteristics of the patients

	*N* = 15
	% (n/N)
Gender	
Male	86.7 (13/15)
Female	13.3 (2/15)
Age	
Median (Min. ‐ Max.)	58 (46–81)
<60 years	53.3 (8/15)
≥60 years	46.7 (7/15)
ECOG PS	
0	13.3 (2/15)
1	80 (12/15)
2	6.7 (1/15)
Karnofsky	
90%	86.7 (13/15)
80%	6.6 (1/15)
70%	6.6 (1/15)
Histology	
Epithelioid	93.3 (14/15)
Other	6.7 (1/15)
Laterality	
Right	66.7 (10/15)
Left	33.3 (5/15)
Tobacco exposure	
Ever‐smoker	53.3 (8/15)
Non‐smokers	46.7 (7/15)
Wood‐smoke exposure	
Yes	6.7 (1/15)
No	93.3 (14/15)
Asbestos exposure	
Yes	73.3 (11/15)
No	26.7 (4/15)

Of the 15 patients that received the three components of multimodality therapy, 10 received induction chemotherapy with cisplatin plus gemcitabine (66.7%), cisplatin plus pemetrexed was used in four patients (26.7%), and cisplatin plus vinorelbine in one patient (6.6%). The median number of cycles was four (2–6), and the number of cycles for each patient was decided by the attending medical oncologist. Every patient included in the study underwent some sort of P/D after induction chemotherapy; six patients (40%) underwent P/D, five patients (33.3%) underwent partial P/D and four patients (26.7%) underwent extended P/D. Macroscopic complete resection was achieved in 13 patients (86.6%). After a median surgery‐recovery time of 6.7 weeks, hemithoracic pleural IMRT was administered at a median dose of 48.7 Gy (23.4–54 Gy). A total of 14 patients (93.3%) completed the predetermined IMRT plan, and one patient stopped IMRT after 23.4 Gy because of esophageal toxicity (Table 2).

**Table 2:** Treatment characteristics and pattern of recurrence

Treatment characteristics	% (n/N)
Chemotherapy regimen	
Cisplatin‐gemcitabine	66.7 (10/15)
Cisplatin‐pemetrexed	26.7 (4/15)
Cisplatin‐vinorelbine	6.6 (1/15)
Pleural/decortication	
Extended P/D	4 (26.7%)
P/D	6 (40%)
Partial P/D	5 (33.3%)
Macroscopic complete resection	
Yes	86.6 (13/15)
No	13.4 (2/15)
Initiated IMRT	
Yes	100 (15/15)
Completed IMRT treatment	
Yes	93.3 (14/15)
No	6.7 (1/15)
Pattern of recurrence	
Local	27.2 (3/11)
Distance	72.7 (8/11)

During the follow‐up period, 11 patients (73.3%) had a documented recurrence, and the predominant pattern of recurrence was distant in eight patients and local in three patients (Table 2).

**Figure 3:**
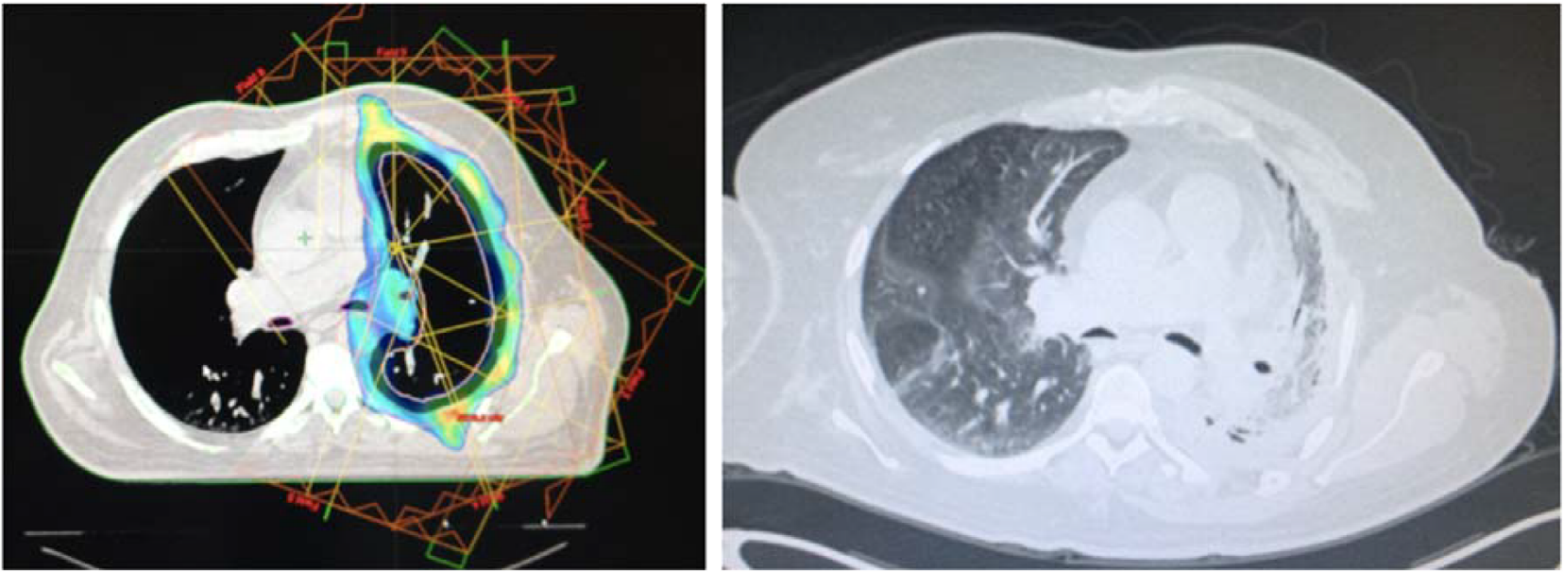
Images from a patient that developed grade 3 radiation pneumonitis, with subsequent pulmonary fibrosis. The patient required steroids and was dependent on oxygen supplementation for eight months once radiation treatment had ended.

A total of nine patients (60%) developed radiation pneumonitis (RP), and of these, five patients (33.3%) had no related symptoms and no further intervention was required. Grade 3–4 RP developed in two patients, and those patients required supplementary oxygen and steroids (Fig 3) (Table 3). Other relevant toxicities were esophagitis and fatigue; esophagitis occurred in six patients (40%), and of these, two patients (13.3%) developed grade three esophagitis. With regard to fatigue, six patients (40%) reported any grade of fatigue, and three patients (20%) reported grade 3 fatigue. No treatment‐related deaths or grade 5 adverse events were documented. A complete recovery to pretreatment‐status was documented in every patient during the follow‐up period.

**Table 3:** Adverse events frequency and grade

	Grade 0	Grade 1–2	Grade 3–4
Adverse event	% (n/N)	% (n/N)	% (n/N)
Pneumonitis	40 (6)	46.6 (7)	13.3 (2)
Esophagitis	60 (9)	26.6 (4)	13.3 (2)
Fatigue	60 (9)	20 (3)	20 (3)

**Figure 4:**
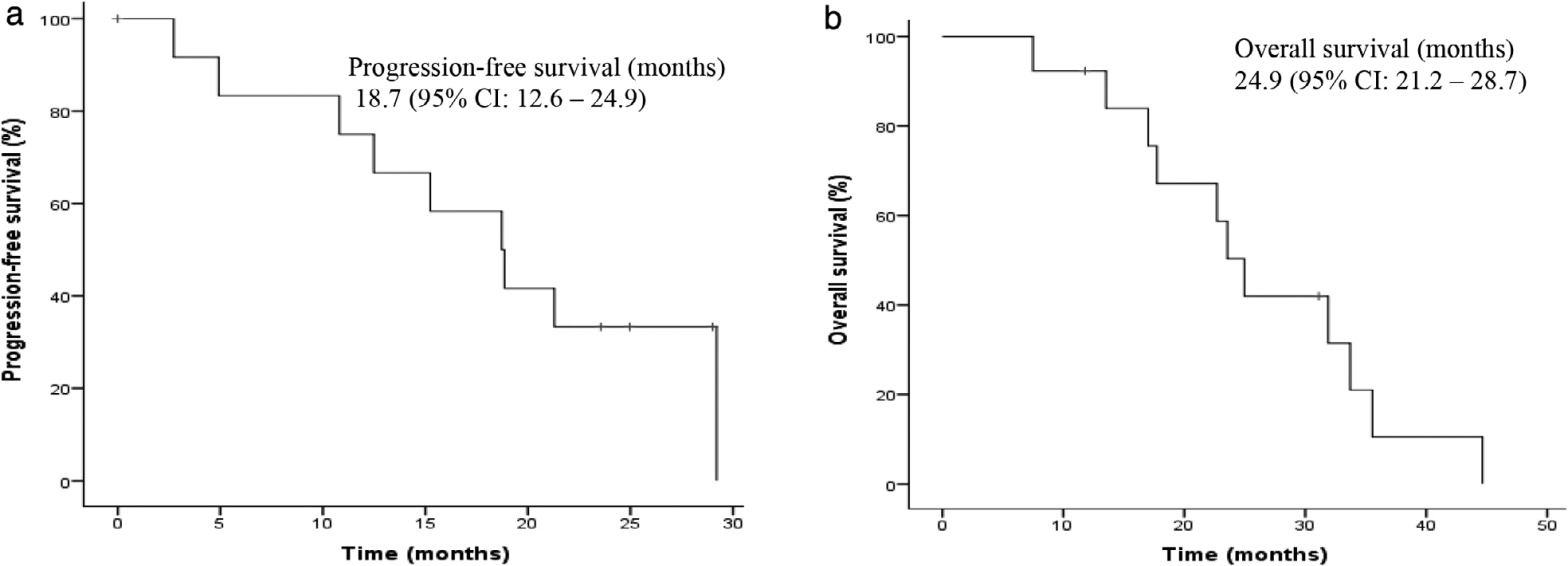
Kaplan‐Meier curves for (**a**) PFS; and (**b**) OS.

The estimated locoregional‐relapse‐free survival at two years was 75.9%. Median PFS was 18.7 months (95% CI: 12.6–24.9). The one‐ and two‐year PFS rates were 72.4% (95% CI: 45.2–88.6) and 26.7% (95% CI: 7.5–51.1), respectively (Fig 4a).

**Figure 5:**
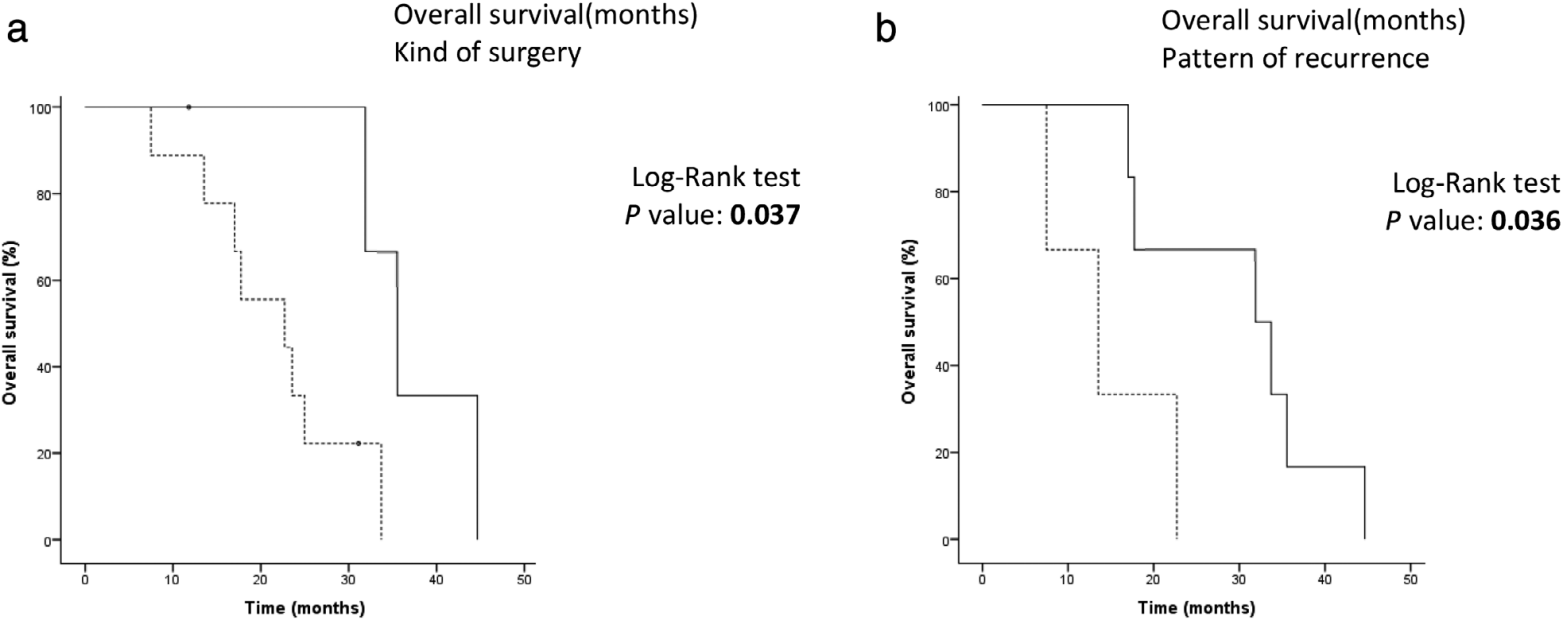
Kaplan‐Meier curve for OS according to (**a**) kind of surgery 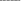, Partial P/D: 22.7 (95% CI: 8.2–37.2); 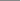, Extended P/D: 35.5 (95% CI: 29.7–41.4); and (**b**) disease recurrence pattern 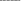, Local: 20.9 (95% CI: 13.5–22.7); 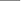, Distant: 31.9 (95% CI: 12.7–51.1).

The median OS was 24.9 months (95% CI: 20.9–26.2) (Fig 4b), one‐ and two‐year OS rates were 86.2% (95% CI: 55.2–96.4) and 50.7% (95% CI: 21.4–71.5), respectively. Patients who underwent an extended P/D also had a favorable median OS compared to those who experienced a partial P/D (35.5 vs. 22.7 months; *P* = 0.037) (Fig 5a). Furthermore, patients with distant disease recurrence achieved longer OS when compared with patients who developed a locoregional recurrence (31.9 vs. 20.9 months; *P* = 0.036) (Fig 5b).

A univariate analysis was performed to analyze PFS and OS according to baseline characteristics and induction‐chemotherapy employed; we did not find any baseline characteristic or chemotherapy regimen significantly associated with differences in PFS or OS (Table 4).

**Table 4:** Univariate analysis for PFS and OS according to baseline characteristics and chemotherapy treatment

	Progression‐free survival		Overall survival	
	Median (95% CI) months	*P*‐value	Median (95% CI) months	*P*‐value
Overall	18.9 (11.8–25.9)		23.6 (20.9–26.2)	
Gender				
Male	18.7 (9.2–28.2)		22.7 (14.5–34.9)	
Female	18.9 (NR)	0.580	23.6 (NR)	0.561
Age				
<60 years	29.2 (NR)		22.1 (13.1–31.2)	
≥60 years	15.2 (5.7–24.7)	0.183	22.7 (9.1–36.2)	0.344
Tobacco exposure				
Current or former smoker	12.5 (6.3–18.6))		17.7 (10.7–24.8)	
Non‐smokers	21.3 (16.1–26.5)	0.471	31.9 (23.1–40.7)	0.590
Wood‐smoke exposure				
Present	18.9 (NR)		35.5 (NR)	
Absent	18.8 (8.5–28.9)	0.738	22.7 (15.9–29.5)	0.396
Asbestos exposure				
Present	18.7 (8.6–28.9)		23.6 (13.8–33.3)	
Absent	18.9 (NR)	0.648	22.1 (15.0–29.2)	0.968
Chemotherapy				
Carboplatin‐pemetrexed	NA		22.1 (NR)	
Cisplatin‐gemcitabine	NA	0.591	24.9 (14.1–35.9)	0.897
Cisplatin‐vinorelbine	NA		23.6 (NR)	

## Discussion

Previous studies have demonstrated that the effectiveness of radiotherapy in the context of a trimodality therapy resulted in a median OS of up to 29.1 months in patients who completed all three modalities of treatment.^2^ In addition, retrospective data also supports this approach, with a study reporting a median OS of 59 months in patients without mediastinal node involvement who completed every component of trimodality therapy.^8^

Although the real benefit of EPP remains controversial; several groups have reported equal or better results with lung‐sparing surgery P/D compared to EPP; this has been reported to be constant in the setting of multimodality therapy.^9^, ^10^, ^11^ Allen *et al*. reported 46.1% of radiotherapy‐related deaths in patients who received IMRT after EPP, with a V20 limited to 20% and a mean lung dose (MLD) of 15 Gy.^12^ Moreover, in a multicenter phase 2 study trial, postoperative hemithoracic high‐dose radiotherapy in the context of trimodality therapy failed to significantly improve locoregional relapse‐free survival compared with no further treatment after EPP. The median locoregional relapse‐free survival in patients that received 3D‐CRT or IMRT was 9.4 months versus 7.6 months for patients who did not receive any sort of RT.^13^

Lung‐sparing techniques (ie, P/D) as surgical treatment of patients with MPM have been associated with a considerably increased risk of local recurrence when used as the only treatment. Furthermore, attempts to reduce locoregional disease relapse with conventional radiation techniques have not yielded optimal results.^14^, ^15^ These results underscore the importance of evaluating IMRT in the multimodal treatment of MPM.

In this study we evaluated efficacy and safety of a trimodality therapy, including platinum‐based induction chemotherapy, lung‐sparing surgery P/D and hemi‐thoracic IMRT. Of the 15 patients that received trimodality therapy, 14 (70%) fully completed the three modalities of treatment. Together, these trimodality therapies led to an excellent locoregional relapse‐free survival of 75.9% at two years, with an acceptable rate of G3‐4 RP (13.3%), and no treatment‐related deaths.

In a previous study, Rosenzweig *et al*. explored adjuvant IMRT in patients who were unable to undergo EPP but who underwent P/D; their results were similar to our results, as they reported a median OS of 26 months for the patients that received IMRT after P/D.^9^ The IMPRINT study, which was the first study to evaluate the feasibility of trimodality therapy involving P/D and IMRT, reported that in patients who completed the pre‐established IMRT, the median PFS was 12.4 months and OS 23.7 months.^1^

At least two studies have explored the role of simultaneous integrated boost SIB in the context of trimodality therapy. A phase 1/2 study demonstrated a median OS and PFS of 28.4 and 16.4 months, respectively in patients who underwent P/D followed by adjuvant IMRT at a dose of 45 Gy in 25 daily fractions; SIB was administered at 60 Gy in high‐risk areas.^10^ In a second study, the dose prescribed was 50 Gy in 25 fractions, and FDG‐avid areas or regions of concern for the residual disease were given a simultaneous boost at 60 Gy. The median OS and PFS were 33 and 29 months, respectively.^16^ Both studies obtained slightly better results than ours in terms of OS, and this could be explained by the potential additive benefit obtained with SIB.

The rates of all grade, and grade ≥3 RP were higher in our study, 60% and 13.3%, respectively, compared with 29.6% and 7.4% reported in the IMPRINT study. One of the most reasonable explanations is related to the dose constraints employed in our study, since we attempted to be extremely strict with the contralateral dose constraints; MLD <3.5 Gy and a V7 < 20%. However, no special considerations were taken in our study for the ipsilateral lung or combined lung dose constraints. A dosimetric analysis identified the contralateral lung dose as a major predictive factor for potentially fatal pneumonitis.^17^ Other potential explanations are the absence of a predefined normal tissue complication probability (NTCP) value; moreover, no additional imaging studies apart from a CT scan were obtained in our study.

There were limitations in our study. First, the small sample size should be considered as a limitation; however, owing to the relatively small number of patients that are suitable for trimodality therapy, prospective studies which involve more patients are rarely seen. Another limitation was that in our study pulmonary function tests (PFTs) were not performed, and therefore we do not know precisely if our treatment approach produced any detrimental effect in lung function. A previous study reported a statistically significant decline of around 25%–31% from baseline PFTs (forced vital capacity [FVC], forced expiratory volume in one second [FEV1], and diffusing capacity of the lung for carbon monoxide [DLCO]) after IMRT treatment.^10^

Development of new highly conformal radiotherapy techniques, such as IMRT, have enabled radiation oncologists to optimize the delivery of high‐dose radiotherapy to the whole hemithorax which is of great utility when treating patients with mesothelioma.^18^ In addition, there is a trend for surgical techniques favoring P/D over EPP. The rationale to deliver adjuvant radiotherapy is therefore increasing, considering the decreased probability of achieving a complete surgical resection after P/D.^19^

In conclusion, the present study reinforces the existing evidence of the role of IMRT after lung‐sparing surgery in the context of trimodality therapy for patients with MPM; in this regard, our results allow us to conclude that trimodality therapy IMRT after P/D provides a better prognosis for patients with locally advanced MPM. Further analysis, ideally multicentric randomized trials with a greater number of patients, are necessary to consolidate this treatment strategy as an option for managing patients with locally advanced MPM.

## Disclosure

The authors report no conflict of interest.

## Supporting information


**Table S1.** Radiation dose constraints for IMRT.
