# Supplementary material for: Locoregional control and toxicity after pleurectomy/decortication and intensity‐modulated pleural radiation therapy in patients with malignant pleural mesothelioma
Source: Thorac Cancer. 2020 Oct 8;11(12):3448–55. doi: 10.1111/1759-7714.13668 (PMC7705616; doi:10.1111/1759-7714.13668)
Supplement: Supplementary file 1 — Table S1. Radiation dose constraints for IMRT. [file TCA-11-3448-s001.docx]

**Supplementary Tables and Figures**

**Supplementary Table 1**. Radiation dose constraints for IMRT

| - Contralateral lung mean dose <3·5 Gy, V7 <20%. |
| --- |
| - Heart: V15 <40% for right-sided tumors. - V18 <70% for left-sided tumors. |
| - Spinal cord D max <22Gy. |
| - Esophagus maximum point dose <30 Gy. |
| - Liver: V17 <60% for right-sided tumors. - V8 <30% for left-sided tumors. |
| - Kidney mean dose <5 Gy, V7 <33 |
